# Validation of the short forms of the Pelvic Floor Distress Inventory and the Pelvic Floor Impact Questionnaire in Estonian

**DOI:** 10.1007/s00192-023-05532-2

**Published:** 2023-04-17

**Authors:** Iveta Mikeltadze, Katrin Täär, Ülle Kadastik, Pille Soplepmann, Kristiina Rull

**Affiliations:** 1 Department of Surgical and Gynecological Oncology, Tartu University Hospital, Puusepa Street 8, 50406 Tartu, Estonia; 2 Institute of Clinical Medicine, University of Tartu, Puusepa Street 8, 50406 Tartu, Estonia; 3 Women’s Clinic of Tartu University Hospital, Puusepa Street 8, 50406 Tartu, Estonia

**Keywords:** Validation study, Pelvic organ prolapse, Pelvic floor, Translations

## Abstract

**Introduction and hypothesis:**

Pelvic Floor Distress Inventory (PFDI-20) and the Pelvic Floor Impact Questionnaire (PFIQ-7) are reliable instruments for evaluating the quality of life in women with pelvic organ prolapse (POP). They have been translated and validated in many languages. The study was aimed at validating the Estonian translations of the PFDI-20 and PFIQ-7 tools.

**Methods:**

The questionnaires were translated into Estonian using a multistep translation method. A total of 132 women were enrolled: patients with diagnosed POP (*n*=57) were allocated to test–retest reliability analyses, and those with no POP signs (*n*=88) completed the questionnaire only once. The total scores of questionnaires and their subscales of both patient and reference groups were compared. Item response rate, floor and ceiling effects, corrected item–total correlations, internal consistency, and convergent and discriminant validity were analyzed. The study was approved by the Ethics Committee of Human Research of the University Clinic of Tartu, Estonia, and informed consent was obtained from each participant.

**Results:**

The translated questionnaires demonstrated good internal consistency (Cronbach's α values 0.77–0.93). The item response rate was 99%. Intra-class correlations (ICC) were strong for PFDI-20 and PFIQ-7 and their subscales ranged from 0.86 to 0.96. Construct validity of the tools demonstrated by manyfold higher scores among patients with POP compared with women without POP (*p*<0.0001).

**Conclusions:**

The Estonian versions of the PFDI-20 and PFIQ-7 tools are reliable and valid instruments for assessing the quality of life in women with POP.

**Supplementary information:**

The online version contains supplementary material available at 10.1007/s00192-023-05532-2.

## Introduction

Pelvic organ prolapse (POP) is a gynecological pathological condition with descent of at least one of the vaginal walls to or beyond the vaginal hymen with maximal Valsalva effort with the presence of either bothersome characteristic symptoms or functional or medical compromise due to prolapse without symptoms [1]. The symptoms of POP can include a vaginal bulge or protrusion, vaginal or pelvic pressure, discomfort or pain in the pelvic area, difficulty emptying the bladder or bowel, urinary incontinence, sexual dysfunction, etc. [2]. The impact of these symptoms can be significant, affecting women's quality of social, psychological, and sexual life [3].

According to available evidence, the prevalence of POP varies widely (1–65%) based on whether its presence is ascertained by symptoms (1–31%), pelvic examination (10–50%), or both (20–65%) [4]. The considerable variation is mainly caused by the lack of uniformity for the definition of POP in epidemiological studies [4]. Also, it is unclear how many women with POP-caused complaints do not have information about their condition/disease and believe it to be a normal, age-related physiological change [5].

Conservative and surgical management of the POP should include the assessment of anatomical changes in the pelvic floor organs and also their effect on the woman's quality of life [6]. Subjective assessments of POP symptoms alone can be unreliable, as patient reports may vary depending on cultural and language differences, individual perception, and reluctance to discuss sensitive issues.

The Pelvic Floor Distress Inventory (PFDI) and Pelvic Floor Impact Questionnaire (PFIQ) [7] were developed in 2001 and have proven to be reliable instruments for evaluating the quality of life in women with POP. The PFDI is used to assess the extent of POP symptoms and related complaints. The PFIQ helps to assess the effect of POP on quality of life. As the questionnaires are very long, the short versions PFDI-20 and PFIQ-7 were compiled, validated, and are now more widely used [8]. They have been translated into different languages and validated in clinical and research settings in many countries [9–21].

Currently, there are no validated tools in Estonian to assess the effect of POP on women's quality of life. Translated and validated questionnaires allow the standardization of the clinical assessment of POP patients, facilitate planning of the optimal management, and help to evaluate the effect of intervention. Validated questionnaires are a prerequisite to carrying out epidemiological studies and participating in international surveys [22].

This study is aimed at validating the Estonian translations of the PFDI-20 and PFIQ-7 tools for clinical assessment of POP-related symptoms and quality of life.

## Materials and methods

The PFDI-20 includes three subscales: the Pelvic Organ Prolapse Distress Inventory (POPDI-6), focusing on prolapse-induced complaints; the Colorectal-Anal Distress Inventory (CRADI-8), assessing defecation disorders; and the Urinary Distress Inventory (UDI-6), addressing urinary complaints caused by POP [8].

The PFIQ-7 similarly has three subscales: the Urinary Impact Questionnaire (UIQ-7); the Colorectal-Anal Impact Questionnaire (CRAIQ-7); and the Pelvic Organ Prolapse Impact Questionnaire (POPIQ-7) [8].

The questionnaires, originally published by Barber et al. [8], were translated into Estonian using a multistep translation method and the test–retest method was applied to validate the questionnaires.

The first step was translating the PFDI-20 and PFIQ-7 by four gynecologists fluent in English and with experience in urogynecology and/or sexual counseling. The four translated versions of each questionnaire were distributed to a group of seven middle-aged to elderly women with a level of completed education varying from primary to higher. Each participant chose the best translation for each question or suggested alternative wording.

Next, the working group consisting of the four gynecologists who translated the questionnaires and two additional gynecologists revised all versions and the participants' answers. As a result of the process, one translated version was compiled. The Estonian version was back-translated into English by a professional medical translator. The original and back-translated English questionnaires were compared and the final version of the PFDI-20 and PFIQ-7 in Estonian were confirmed.

The final version of the Estonian PFDI-20 and PFIQ-7 questionnaires were distributed to patients at the outpatient department at the Women's Clinic of Tartu University Hospital in 2020–2022. Tartu University Hospital is a tertiary-level hospital that serves as a referral center for patients with POP–related problems. In addition, owing to its close cooperation with the University of Tartu, the hospital is a leading center for medical research and education in Estonia.

Only patients who were fluent in the Estonian language could complete the questionnaire independently, and signed informed consent forms to participate were included in the study. Patients with malignant diseases or unexplained bleeding from the genital tract or with indications for immediate treatment, such as insertion of a vaginal pessary or surgical repair, were excluded from the study. Patients with diagnosed and at least one sign of POP were defined as the patient group and allocated to test–retest reliability analyses, and those with no POP signs were classified as the reference group.

Patients with POP were asked to complete and return the same PFDI-20 and PFIQ-7 in 2 weeks. Asymptomatic women completed the questionnaires only once. Based on the focus of the study only basic data of the participants were collected: age and presence of POP assessed by gynecologist or midwife.

The study was approved by the Ethics Committee of Human Research of the University Clinic of Tartu, Estonia (permissions no. 302/T-16 16.03.2020, 319/M-19 15.06.2020, and 340/M-30 19.04.2021, 352/T-9 15.11.2021), and informed consent was obtained from each participant.

The PFIQ-7 and PFDI-20 were tested for construct validity and reliability. Average scores with standard deviations as well as minimum and maximum values in each scale were reported. The overall response rate among the patient group was defined as the proportion of the patients who returned the two questionnaires in 2 weeks. The item response rate was defined as the proportion of questions answered in each questionnaire. Reliability was assessed by test–retest analysis. The correlation between the answers given at the two different time points was assessed using the intra-class correlation coefficient (ICC). Internal consistency was measured with Cronbach's α. Cronbach's α values <0.7 were considered too heterogeneous, >0.95 too similar, and values of 0.7–0.95 were considered to have adequate internal consistency.

For testing construct validity, item response rate, floor and ceiling effects (people with minimum and maximum scores respectively), and corrected item–total correlations were analyzed. Corrected item–total correlations ≥0.3 were considered acceptable and evidence of convergent validity. Spearman's rank-order correlation was used to assess convergent and discriminant validity, analyzing intercorrelation between the subscales and between different tools.

For assessment of clinical validity, the PFIQ-7 and PFDI-20 questionnaires were filled out by the women with and without POP. The total scores of questionnaires and their subscales of both groups were compared. The STATA software version 13.1 (StataCorp, College Station, TX, USA) was used for data analysis.

For the translated questionnaire documents: see Appendices S1, S2.

## Results

**Fig. 1:**
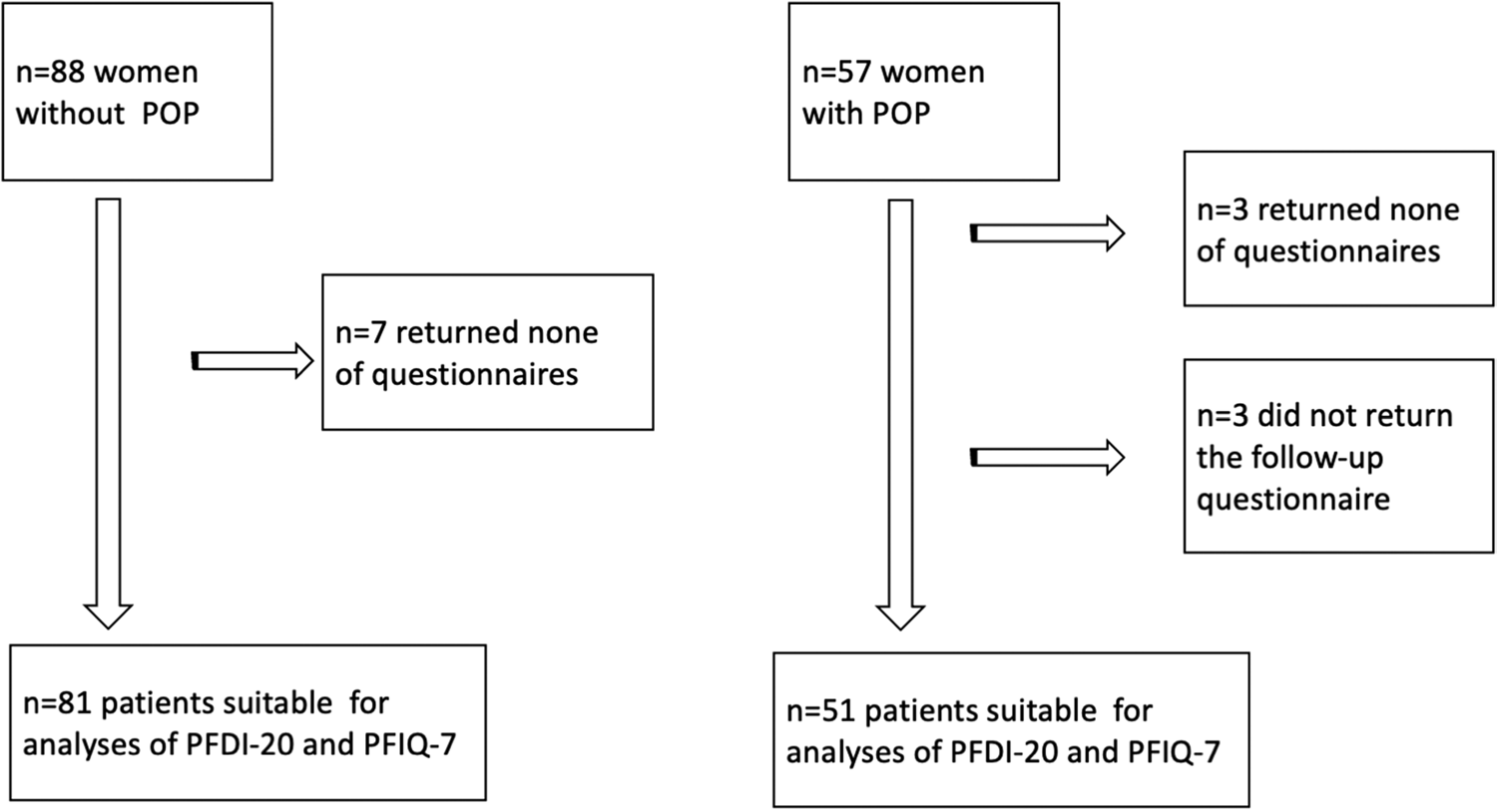
Flowchart of patients without pelvic organ prolapse (*POP*) and with POP allocated for validation of the Pelvic Floor Distress Inventory (*PFDI-20*) and Pelvic Floor Impact Questionnaire (*PFIQ-7*)

Fifty-seven patients with a POP diagnosis were recruited for validation of the PFIQ-7 and PFDI-20 (Fig. 1). Three patients did not return any of the questionnaires, and 3 did not return the follow-up questionnaire sent 2 weeks after the initial questionnaire. Only data from the 51 patients who completed both questionnaires were included in the data analysis (Fig. 1). The overall response rate for PFIQ-7 and PFDI-20 was 91.1%. The item response rate was 99%.

Seven patients out of the 88 women without POP signs did not return any of the PFIQ-7 and PFDI-20 and were excluded from the analysis (Fig. 1). The overall response rate was 92%.

The mean age of the POP patients was 53.9±11.6 years and 48±9.1 years among women without POP. All age groups from 30 to 75 were represented in both groups.

Among POP patients, the floor effect exhibiting the minimum value of zero with subscales of PFIQ-7 ranged between 7.8 and 29.4%. No floor effects were observed in the PFDI-20 and PFIQ-7 total scores. No ceiling effects were observed with any of the questionnaires (Table 1).

**Table 1:** The prevalence of floor and ceiling effects of baseline score among 51 women diagnosed with pelvic organ prolapse

Questionnaire and subscales (score range in points)	Floor effect, *n* (%)	Ceiling effect, *n* (%)
PFDI-20 (0–300)	0 (0)	0 (0)
POPDI-6 (0–100)	0 (0)	0 (0)
CRADI-8 (0–100)	1 (2)	0 (0)
UDI-6 (0–100)	0 (0)	0 (0)
PFIQ-7 (0–300)	0 (0)	0 (0)
POPIQ-7 (0–100)	15 (29.4)	0 (0)
CRAIQ-7 (0–100)	15 (29.4)	0 (0)
UIQ-7 (0–100)	4 (7.8)	0 (0)

The floor effect reflects the lower limit of the score and the ceiling effect reflects the upper limit of the score

*PFDI* Pelvic Floor Distress Inventory, *POPDI* Pelvic Organ Prolapse Distress Inventory, *CRADI* Colorectal-Anal Distress Inventory, *UDI* Urinary Distress Inventory, *PFIQ* Pelvic Floor Impact Questionnaire, *POPIQ* Pelvic Organ Prolapse Impact Questionnaire, *CRAIQ* Colorectal-Anal Impact Questionnaire, *UIQ* Urinary Impact Questionnaire

In the test–retest analysis intra-class correlations (ICC) were strong for PFIQ-7 and PFDI-20 and their subscales, ranging from 0.86 for the UDI-6 to 0.96 for the UIQ-7 (*p*<0.0001; Table 2).

**Table 2:** Intra-class correlation and internal consistency among the patients diagnosed with pelvic organ prolapse for evaluating the reliability of the questionnaires

	Intraclass correlation (95% CI)	Cronbach's α	
		Test	Retest
PFIQ-7	0.96 (0.93–0.98)	0.93	0.94
UIQ-7	0.96 (0.93–0.98)	0.91	0.93
CRAIQ-7	0.93 (0.87–0.95)	0.94	0.95
POPIQ-7	0.95 (0.92–0.98)	0.92	0.93
PFDI 20	0.94 (0.89–0.96)	0.85	0.85
POPDI-6	0.90 (0.84–0.94)	0.76	0.77
CRADI-8	0.92 (0.86–0.95)	0.83	0.85
UDI-6	0.86 (0.77–0.92)	0.53	0.64

*CI* confidence interval, *PFIQ* Pelvic Floor Impact Questionnaire, *UIQ* Urinary Impact Questionnaire, *CRAIQ* Colorectal-Anal Impact Questionnaire, *POPIQ* Pelvic Organ Prolapse Impact Questionnaire, *PFDI* Pelvic Floor Distress Inventory, *POPDI* Pelvic Organ Prolapse Distress Inventory, *CRADI* Colorectal-Anal Distress Inventory, *UDI* Urinary Distress Inventory

The internal consistency for the PFIQ-7 and its subscales (UIQ-7, CRAIQ-7, and POPIQ-7) was good (Cronbach's α values 0.91–0.95). The PFDI-20 and its subscales POPDI-6 and CRADI-8 also showed sufficient internal consistency (Cronbach's α values 0.76–0.85). The subscale UDI-6 indicated higher heterogeneity, with Cronbach's α values of 0.53 and 0.64 (Table 2).

The corrected item–total correlations demonstrated that the PFIQ-7 had acceptable construct validity. The correlations were *r*=0.608–0.863 for the UIQ-7, *r*=0.733–0.891 for the CRAIQ-7, *r*=0.597–0.873 for the POPIQ-7, and *r*=0.526–0.810 for the total tool (PFIQ-7; Supplementary Table 1). PFDI-20 item–total correlations for the total score were lower, *r*=0.010–0.684, five questions had *r*<0.3 (Supplementary Table 2). One question in the POPDI-6 and one in CRADI-8 subscales demonstrated a weak correlation *r*<0.3 with the total score. Other questions had moderate or high correlation (*r*=0.489–0.692 and *r*=0.465–0.739 respectively; Supplementary Table 2). In concordance with the lower internal consistency of UDI-6, its questions had a weaker item–total correlation. The lowest convergent validity was observed with the UDI-6 with *r*=0.0472–0.4521 (Supplementary Table 2).

Convergent validity analyses showed strong correlation between PFIQ-7 and PFDI-20 total scores (*r*=0.7830) as well as correlation between respective subscales (*r*=0.5592–0.6467) among patients with POP (Table 3). The convergent validity of the PFIQ-7 and PFDI-20 was considered sufficient.

**Table 3:** Convergent validity of the tools, including their subscales

	UIQ-7	CRAIQ-7	POPIQ-7	PFDI-20	POPDI-6	CRADI-8	UDI-6
CRAIQ-7	0.2678						
POPIQ-7	0.3939*	0.5496*					
PFDI-20	0.4467*	0.7296*	0.6267*				
POPDI-6	0.2675	0.5164*	0.7384*	0.8038*			
CRADI-8	0.1767	0.7323*	0.3599*	0.8280*	0.5425*		
UDI-6	0.8168*	0.3190*	0.3342*	0.6021*	0.2937*	0.2598	
PFIQ-7	0.7132*	0.7822*	0.7590*	0.7830*	0.6399*	0.5592*	0.6467*

Spearman correlation coefficients are given; *p<0.05

*CRAIQ* Colorectal-Anal Impact Questionnaire, *POPIQ* Pelvic Organ Prolapse Impact Questionnaire, *PFDI* Pelvic Floor Distress Inventory, *POPDI* Pelvic Organ Prolapse Distress Inventory, *CRADI* Colorectal-Anal Distress Inventory, *UDI* Urinary Distress Inventory, *PFIQ* Pelvic Floor Impact Questionnaire

The construct validity of the questionnaires was demonstrated by comparing the total score and subscales of the PFDI-20 (POPDI-6, CRADI-8, UDI-6), the PFIQ-7 (UIQ-7, CRAIQ-7, POPIQ-7) in patients with POP and women without POP. All scores were manyfold higher among patients at both time points than among women who did not present any signs of POP (*p*<0.0001, Table 4).

**Table 4:** Comparison of total and subscale scores of all questionnaires assessing the complaints caused by pelvic floor prolapse among women with and without pelvic organ prolapse (*POP*)

	Mean score (SD)			*p* value^a^
	With POP, *n* = 51		Without POP, *n* = 81	
	Test	Retest*		
PFIQ-7	90.5 (68.1)	89.7 (67.9)	19.9 (28.7)	<0.0001
UIQ-7	42.1 (28.2)	42.7 (29.5)	11.1 (16.5)	<0.0001
CRAIQ-7	24.9 (29.1)	24.3 (27.2)	7.1 (14.3)	<0.0001
POPIQ-7	22.6 (27.4)	22.4 (24.4)	1.7 (6.3)	<0.0001
PFDI-20	143.7 (44.2)	140.2 (47.9)	61.5 (41.6)	<0.0001
POPDI-6	47.0 (20.5)	46.7 (19.7)	16.1 (13.9)	<0.0001
CRADI-8	43.0 (21.8)	42.7 (21.2)	21.9(16)	<0.0001
UDI-6	53.7 (16.4)	53.3 (17.2)	23.5 (16.8)	<0.0001

*SD* standard deviation, *PFIQ* Pelvic Floor Impact Questionnaire, *UIQ* Urinary Impact Questionnaire, *CRAIQ* Colorectal-Anal Impact Questionnaire, *POPIQ* Pelvic Organ Prolapse Impact Questionnaire, *PFDI* Pelvic Floor Distress Inventory, *POPDI* Pelvic Organ Prolapse Distress Inventory, *CRADI* Colorectal-Anal Distress Inventory, *UDI* Urinary Distress Inventory,

^*^Mean scores were not statistically different between all questionnaires filled in at 2-week intervals (test–retest)

^a^Comparison was made between the mean score of the first questionnaire (test) filled out by patients and asymptomatic women

## Discussion

The aim of the present study was to validate the Estonian translations of the PFDI-20 and PFIQ-7 tools used to evaluate symptoms and quality of life associated with POP. Both instruments demonstrated good reliability and validity, allowing recommend their application in clinical practice to be recommended. The usage of PDFI-20 and PFIQ-7 enables more accurate clinical evaluation and effective treatment of women with POP-related symptoms, providing clinicians and researchers with a valuable resource for future studies in this field.

Pelvic organ prolapse and its complications are associated with poorer quality of social, psychological, and sexual life [3]. In clinical practice, problems related to urinary, defecatory, and vaginal dysfunction are often underestimated. Women may feel ashamed to talk about POP and its symptoms. Self-filled questionnaires are valuable tools for realizing and describing the severity of problems caused by POP and help to assess the patient's complaints.

In clinical questionnaires, the floor and ceiling effect, or a number of subjects with minimum or maximum scores, is greatly influenced by the clinical condition the questionnaire focuses on and health status. Therefore, had these questionnaires been answered by individuals with no POP or, in contrast, by patients with extreme prolapse, the results could have been influenced by their extreme scores. To minimize the floor and ceiling effect, the current study included individuals at different POP stages.

In both the present study and a previous study validating the Finnish version of the PFIQ-7 [14], a floor effect was observed in the PFIQ-7 subscales. However, in the Finnish version, in contrast to the current study, the floor effect was also observed in the total scale. The Danish version of the same questionnaire [23] reported the opposite finding of a significant ceiling effect. As the PFIQ-7 tool reflects how the patient's bowel, bladder, and/or pelvic symptoms affect their daily life, social relationships, and emotional well-being, the observed variations between studies may indicate cultural and social differences between countries.

The results of the PFDI-20 measurement properties, addressed in the current study, were consistent with those of several other translation and validation studies [24]. The internal consistency of the PFDI-20 tool was satisfactory, with good reproducibility across 2 weeks for its subscales POPDI-6 and CRADI-8, as indicated by the ICC. However, the current study observed higher heterogeneity in the UDI-6 subscale, which measures the severity of urinary distress, such as frequent urination and urine leakage, compared with other studies [24]. Nevertheless, similar high heterogeneity in the UDI-6 subscale was also reported in the Turkish [25], Finnish [14], Spanish [16], and Dutch [19] versions. It is possible that women consider urinary complaints as normal physiological changes associated with aging and previous pregnancies and tend not to report them. However, after receiving counseling from a doctor and being asked the same questions in the UDI-6 subscale 2 weeks later, they may become more aware of the pathological condition and report symptoms more easily, leading to an increase in the internal consistency of the UDI-6 subscale.

The strength of the current study is the high response rate for all questionnaires. The recruiting doctors paid a lot of attention to explaining the need to complete the questionnaires. A minimal number of unanswered questions made it easier to analyse the data. As the age of the patients recruited ranged from 30 to 75 years, the validated tools can be used at different ages. The inclusion of the asymptomatic women demonstrated significant differences in the total score and subscales of both tools, showing adequate validity of the instruments.

The small sample size and single-center involvement are the major limitations of the current study, but the referral status of the university hospital, minimal linguistic and cultural differences among the Estonian-speaking population owing to the small size of the country can weaken the limitations.

In conclusion, this study provides evidence for the reliability and validity of the Estonian versions of the PFDI-20 and PFIQ-7 tools in assessing the extent of POP symptoms and their effect on the quality of life in women with POP. Overall, our findings contribute to the growing body of literature on pelvic floor dysfunction and provide valuable insights for clinicians managing patients with POP in Estonia and beyond.

## Supplementary information

Below is the link to the electronic supplementary material.

Supplementary file1 (DOCX 36 KB)
