# Supplementary material for: Validation of the short forms of the Pelvic Floor Distress Inventory and the Pelvic Floor Impact Questionnaire in Estonian
Source: Int Urogynecol J. 2023 Apr 17;34(9):2235–40. doi: 10.1007/s00192-023-05532-2 (PMC10506922; doi:10.1007/s00192-023-05532-2)
Supplement: Supplementary file 1 — Supplementary file1 (DOCX 36 KB) [file 192_2023_5532_MOESM1_ESM.docx]

Supplementaray Material

**Validation of the short forms of the Pelvic Floor Distress Inventory (PFDI-20) and Pelvic Floor Impact Questionnaire (PFIQ-7) in Estonian**

Iveta Mikeltadze, Katrin Täär, Ülle Kadastik, Pille Soplepmann, Kristiina Rull

List of supplementary materials:

**Appendix S1**: Vaagnapõhja funktsioonihäire indeks, PFDI-20 (Pelvic Floor Distress Inventory)

**Appendix S2**: Küsimustik vaagnapõhja häirete mõju hindamiseks igapäevaelus, PFIQ-7 (Pelvic Floor Impact Questionnaire )

**Supplementary Table S1**: Item-total correlations for PFIQ-7 and its subscales

**Supplementary Table S2**: Item-total correlations for PFDI-20 and its subscales

Original questionnaires can be assessed:

Barber M, Walters M, Bump R. Short forms of two condition-specific quality-of-life questionnaires for women with pelvic floor disorders (PFDI-20 and PFIQ-7). Am J Obstet Gynecol. 2005;193(1):103–113.

**Appendix S1**

**Vaagnapõhja funktsioonihäire indeks, PFDI-20 (Pelvic Floor Distress Inventory)**

Palun vastake kõikidele järgnevatele küsimustele.

Küsimuste eesmärk on välja selgitada, kas Teil esineb teatud soole, põie ja vaagnapõhja allavaje sümptomeid ning kuivõrd need mõjutavad Teie elukvaliteeti.

Küsimustele vastates mõelge hinnatavate sümptomite esinemisele viimase 3 kuu jooksul ning tõmmake ring ümber sobivale vastusele.

PFDI-20 küsimustik koosneb 20 küsimusest.

**Sümptomite esinemisel kaebuste ulatus:** 1 = üldse mitte

2 = mõnevõrra

3 = mõõdukalt

4 = suurel määral

**Sümptomite puudumisel:** 0 = puudub

| **Viimase 3 kuu jooksul esinenud sümptomid** | **ei esine**  **0** | **Kui palju need sümptomid häirivad Teie igapäevast elu?** | | | |
| --- | --- | --- | --- | --- | --- |
|  |  | **üldse mitte**  **1** | **mõne-võrra**  **2** | **mõõdu-kalt**  **3** | **suurel määral**  **4** |
| **Vaagnaelundite allavaje häirete hindamine 6 (POPDI-6)** | | | | | |
| 1. Kas esineb surve- või raskustunne alakõhus? | 0 | 1 | 2 | 3 | 4 |
| 2. Kas esineb valu selja alaosas või alakõhus? | 0 | 1 | 2 | 3 | 4 |
| 3. Kas tunnete allavajet või midagi tupest „välja tulemas“? | 0 | 1 | 2 | 3 | 4 |
| 4. Kas peate soole täielikuks tühjendamiseks vajutama tupe või pärasoole piirkonda? | 0 | 1 | 2 | 3 | 4 |
| 5. Kas tunnete et põis ei tühjene täielikult? | 0 | 1 | 2 | 3 | 4 |
| 6. Kas peate allavajet tagasi lükkama selleks, et saaksite urineerida/põit tühjendada? | 0 | 1 | 2 | 3 | 4 |

| **Viimase 3 kuu jooksul esinenud sümptomid** | | **ei esine**  **0** | | **Kui palju need sümptomid häirivad Teie igapäevast elu?** | | | | |  |
| --- | --- | --- | --- | --- | --- | --- | --- | --- | --- |
|  |  |  |  | **üldse mitte**  **1** | | **mõne-võrra**  **2** | **mõõdu-kalt**  **3** | **suurel määral**  **4** |  |
| **Kolorektaal-päraku häirete hindamine (CRAD-8)** | | | | | | | | |  |
| 7. Kas peate soole tühjendamiseks kõvasti punnitama? | | 0 | | 1 | | 2 | 3 | 4 |  |
| 8. Kas tunnete, et pärast roojamist ei ole sool täielikult tühjenenud? | | 0 | | 1 | | 2 | 3 | 4 |  |
| 9. Kas konsistentsilt kõva väljaheite korral on roojapidamine raskendatud? | | 0 | | 1 | | 2 | 3 | 4 |  |
| 10. Kas konsistentsilt vedela väljaheite korral on roojapidamine raskendatud? | | 0 | | 1 | | 2 | 3 | 4 |  |
| 11. Kas esineb tahtmatut soolegaaside pidamatust? | | 0 | | 1 | | 2 | 3 | 4 |  |
| 12. Kas roojamisega kaasneb valu? | | 0 | | 1 | | 2 | 3 | 4 |  |
| 13. Kas roojamisvajaduse korral peate kiiresti jõudma WC-sse? | | 0 | | 1 | | 2 | 3 | 4 |  |
| 14. Kas esineb roojamise ajal või roojamise järgselt soole väljalangust? | | 0 | | 1 | | 2 | 3 | 4 |  |
| **Urineerimishäirete hindamine (UDI-6):** | | | | | | | | | |
| 15. Kas esineb sagenenud urineerimist? | 0 | | 1 | | 2 | | 3 | 4 | |
| 16. Kas urineerimistungi korral kaasneb uriinileke enne tualetti jõudmist? | 0 | | 1 | | 2 | | 3 | 4 | |
| 17. Kas uriinileke esineb köhimisel, aevastamisel või naermisel? | 0 | | 1 | | 2 | | 3 | 4 | |
| 18. Kas uriinileke on tavaliselt väikeste kogustena (mõned tilgad)? | 0 | | 1 | | 2 | | 3 | 4 | |
| 19. Kas esineb raskusi põie tühjendamisega? | 0 | | 1 | | 2 | | 3 | 4 | |
| 20. Kas esineb valu või ebamugavustunnet alakõhus või suguelundite piirkonnas? | 0 | | 1 | | 2 | | 3 | 4 | |

**Appendix S2**

**Küsimustik vaagnapõhja häirete mõju hindamiseks igapäevaelus, PFIQ-7 (Pelvic Floor Impact Questionnaire )**

Märkige iga küsimuse juures ristiga vastus, mis kirjeldab kõige paremini kuivõrd Teie igapäevaelu on mõjutatud Teie põie, soole ja vaagnapõhja allavajest tingitud sümptomite poolt viimase 3 kuu jooksul.

Palun märkige vastus iga küsimuse järel kõigis kolmes tulbas.

| Kuidas järgnevad sümptomid /seisundid mõjutavad Teie alltoodud tegevusi? | Põis/urineerimine  UIQ-7 | Sool/pärasool/  Roojamine  CRAIQ-7 | Vaagen/tupp  POPIQ-7 |
| --- | --- | --- | --- |
| 1. Kodutööde tegemine: näiteks toiduvalmistamine, koristamine, pesu pesemine ja triikimine | 0 üldse mitte  1 mõnevõrra  2 mõõdukalt  3 suurel määral | 0 üldse mitte  1 mõnevõrra  2 mõõdukalt  3 suurel määral | 0 üldse mitte  1 mõnevõrra  2 mõõdukalt  3 suurel määral |
| 2. Füüsiline aktiivsus: treeningud, jalutamine, ujumine, jt | 0 üldse mitte  1 mõnevõrra  2 mõõdukalt  3 suurel määral | 0 üldse mitte  1 mõnevõrra  2 mõõdukalt  3 suurel määral | 0 üldse mitte  1 mõnevõrra  2 mõõdukalt  3 suurel määral |
| 3. Meelelahutuslikud üritused: kinos, teatris, kontserdil käimine | 0 üldse mitte  1 mõnevõrra  2 mõõdukalt  3 suurel määral | 0 üldse mitte  1 mõnevõrra  2 mõõdukalt  3 suurel määral | 0 üldse mitte  1 mõnevõrra  2 mõõdukalt  3 suurel määral |
| 4. Bussi- või autosõit kodust kaugemale, sõidu kestus rohkem kui 30 min | 0 üldse mitte  1 mõnevõrra  2 mõõdukalt  3 suurel määral | 0 üldse mitte  1 mõnevõrra  2 mõõdukalt  3 suurel määral | 0 üldse mitte  1 mõnevõrra  2 mõõdukalt  3 suurel määral |
| 5. Sotsiaalsed tegevused väljaspool kodu | 0 üldse mitte  1 mõnevõrra  2 mõõdukalt  3 suurel määral | 0 üldse mitte  1 mõnevõrra  2 mõõdukalt  3 suurel määral | 0 üldse mitte  1 mõnevõrra  2 mõõdukalt  3 suurel määral |
| 6. Vaimne tervis: närvilisus, depressioon jt. | 0 üldse mitte  1 mõnevõrra  2 mõõdukalt  3 suurel määral | 0 üldse mitte  1 mõnevõrra  2 mõõdukalt  3 suurel määral | 0 üldse mitte  1 mõnevõrra  2 mõõdukalt  3 suurel määral |
| 7. Üldine häiritud olek | 0 üldse mitte  1 mõnevõrra  2 mõõdukalt  3 suurel määral | 0 üldse mitte  1 mõnevõrra  2 mõõdukalt  3 suurel määral | 0 üldse mitte  1 mõnevõrra  2 mõõdukalt  3 suurel määral |

**Supplementary Table S1**: Item-total correlations for PFIQ-7 and its subscales

| PFIQ-7 | *r* | UIQ-7 | *r* | CRAIQ-7 | *r* | POPIQ-7 | *r* |
| --- | --- | --- | --- | --- | --- | --- | --- |
| CRAIQ-7 Q1 | 0.6612 | Q1 | 0.7077 | Q1 | 0.8429 | Q1 | 0.8219 |
| CRAIQ-7 Q2 | 0.7116 | Q2 | 0.7236 | Q2 | 0.7916 | Q2 | 0.8730 |
| CRAIQ-7 Q3 | 0.8095 | Q3 | 0.8245 | Q3 | 0.8907 | Q3 | 0.8071 |
| CRAIQ-7 Q4 | 0.5616 | Q4 | 0.7077 | Q4 | 0.7334 | Q4 | 0.5973 |
| CRAIQ-7 Q5 | 0.7425 | Q5 | 0.8634 | Q5 | 0.7474 | Q5 | 0.7959 |
| CRAIQ-7 Q6 | 0.6351 | Q6 | 0.6082 | Q6 | 0.7787 | Q6 | 0.7128 |
| CRAIQ-7 Q7 | 0.6838 | Q7 | 0.6747 | Q7 | 0.7939 | Q7 | 0.6960 |
| POPIQ-7 Q1 | 0.6491 |  |  |  |  |  |  |
| POPIQ-7 Q2 | 0.670 |  |  |  |  |  |  |
| POPIQ-7 Q3 | 0.7280 |  |  |  |  |  |  |
| POPIQ-7 Q4 | 0.5612 |  |  |  |  |  |  |
| POPIQ-7 Q5 | 0.6980 |  |  |  |  |  |  |
| POPIQ-7 Q6 | 0.5259 |  |  |  |  |  |  |
| POPIQ-7 Q7 | 0.6602 |  |  |  |  |  |  |
| UIQ-7 Q1 | 0.6911 |  |  |  |  |  |  |
| UIQ-7 Q2 | 0.6651 |  |  |  |  |  |  |
| UIQ-7 Q3 | 0.8018 |  |  |  |  |  |  |
| UIQ-7 Q4 | 0.6263 |  |  |  |  |  |  |
| UIQ-7 Q5 | 0.8348 |  |  |  |  |  |  |
| UIQ-7 Q6 | 0.5263 |  |  |  |  |  |  |
| UIQ-7 Q7 | 0.6605 |  |  |  |  |  |  |

* Corrected item-total correlations ≥0.3 were considered acceptable and evidence of convergent validity.

**Q stands for question

**Supplementary Table S2**: Item-total correlations for PFDI-20 and its subscales

| PFDI-20 | *r* | POPDI-6 | *r* | CRADI-8 | *r* | UDI-6 | *r* |
| --- | --- | --- | --- | --- | --- | --- | --- |
| POPDI-6 Q1 | 0.6372 | Q1 | 0.5534 | Q1 | 0.6155 | Q1 | 0.1910 |
| POPDI-6 Q2 | 0.4608 | Q2 | 0.4885 | Q2 | 0.7389 | Q2 | 0.4521 |
| POPDI-6 Q3 | 0.3532 | Q3 | 0.5050 | Q3 | 0.4647 | Q3 | 0.3822 |
| POPDI-6 Q4 | 0.4018 | Q4 | 0.2628 | Q4 | 0.5951 | Q4 | 0.2027 |
| POPDI-6 Q5 | 0.4603 | Q5 | 0.5591 | Q5 | 0.6123 | Q5 | 0.1254 |
| POPDI-6 Q6 | 0.5041 | Q6 | 0.6926 | Q6 | 0.7118 | Q6 | 0.0472 |
| UDI-6 Q1 | 0.2836 |  |  | Q7 | 0.4867 |  |  |
| UDI-6 Q2 | 0.2499 |  |  | Q8 | 0.2476 |  |  |
| UDI-6 Q3 | 0.0099 |  |  |  |  |  |  |
| UDI-6 Q4 | 0.0549 |  |  |  |  |  |  |
| UDI-6 Q5 | 0.3096 |  |  |  |  |  |  |
| UDI-6 Q6 | 0.5717 |  |  |  |  |  |  |
| CRADI-8 Q1 | 0.5098 |  |  |  |  |  |  |
| CRADI-8 Q2 | 0.6837 |  |  |  |  |  |  |
| CRADI-8 Q3 | 0.4474 |  |  |  |  |  |  |
| CRADI-8 Q4 | 0.5477 |  |  |  |  |  |  |
| CRADI-8 Q5 | 0.6423 |  |  |  |  |  |  |
| CRADI-8 Q6 | 0.5086 |  |  |  |  |  |  |
| CRADI-8 Q7 | 0.4738 |  |  |  |  |  |  |
| CRADI-8 Q8 | 0.1578 |  |  |  |  |  |  |

* Corrected item-total correlations ≥0.3 were considered acceptable and evidence of convergent validity.

**Q stands for question
